# Interdental Papilla Morphology in Relation to Periodontal Soft- and Hard-Tissue Dimensions in Young Adults: A Clinical and CBCT-Based Cross-Sectional Study

**DOI:** 10.3390/diagnostics16182937

**Published:** 2026-09-11

**Authors:** Basak Karasu, Zuhal Ovuz, Remzi Orkun Akgun

**Affiliations:** 1 Department of Periodontology, Faculty of Dentistry, Cankiri Karatekin University, Cankiri 18100, Türkiye; 2 Department of Oral and Maxillofacial Radiology, Faculty of Dentistry, Cankiri Karatekin University, Cankiri 18100, Türkiye; zuhalovuz@karatekin.edu.tr; 3 Department of Basic Sciences, Faculty of Dentistry, Cankiri Karatekin University, Cankiri 18100, Türkiye; roakgun@karatekin.edu.tr

**Keywords:** periodontal phenotype, papilla morphology, papilla fill, cone-beam computed tomography

## Abstract

**Background/Objectives**: Interdental papilla morphology is an important component of anterior periodontal esthetics. This study aimed to characterize periodontal phenotype distribution in young adults and investigate the relationships between interdental papilla morphology and gingival and alveolar tissue dimensions in the maxillary central incisor region. **Methods**: A total of 238 maxillary central incisors from 119 periodontally healthy young adults were evaluated. Gingival thickness (GT) was assessed by probe transparency (PT) and CBCT, while buccal bone thickness (BT), keratinized tissue width (KTW), papilla height (PH), and interdental papilla fill status were recorded. Associations between papilla morphology and periodontal tissue dimensions and relationships among soft- and hard-tissue phenotype components were analyzed. **Results**: A thin periodontal phenotype predominated, identified in 54.6% of sites by CBCT-based assessment and 43.6% by PT. GT and BT were significantly greater in thick than thin phenotype groups (*p* < 0.001), while KTW showed a site-specific difference. Papilla fill status was not significantly associated with GT, BT, KTW, or PH (*p* > 0.05), and PH was comparable between phenotype groups. GT was positively correlated with corresponding buccal BT (ρ = 0.410 and 0.521; *p* < 0.001) and, more weakly, with KTW (ρ = 0.204 and 0.216; *p* < 0.05). **Conclusions**: In periodontally healthy young adults, papilla fill status was not associated with GT, BT, KTW, or PH, suggesting that papillary appearance may not directly reflect surrounding periodontal tissue dimensions. In contrast, the positive GT–BT relationship supports a structural link between the soft- and hard-tissue components of the periodontal phenotype.

## 1. Introduction

The term ‘periodontal phenotype’ encompasses the gingival phenotype, which includes gingival thickness (GT) and the width of keratinized tissue (KTW), as well as the bone morphotype, which refers to the thickness of the labial or buccal alveolar crest [1]. Recent studies have highlighted the clinical importance of the gingival phenotype in periodontal, restorative, orthodontic, and implant therapies [2,3,4]. Accurate assessment of soft- and hard-tissue dimensions is essential, as it can influence the outcomes of periodontal therapy, particularly in aesthetically sensitive areas. Buccal bone thickness (BT), as a component of the periodontal phenotype, is also considered an important parameter in treatment planning within the esthetic zone [5]. Therefore, the maxillary anterior region, particularly the maxillary central incisors (CIs), has frequently been analyzed to develop reliable guidelines for identifying critical cases characterized by thin gingiva and/or alveolar bone [6,7,8,9]. From a clinical perspective, gingival phenotypes are generally classified as either thin or thick [4,10,11]. The thin phenotype is associated with increased susceptibility to gingival recession and its associated complications [4,12]. The risk of gingival recession increases during restorative and orthodontic treatments, as well as during implant procedures [13]. Periodontal phenotype may also vary according to individual and anatomical characteristics, including sex, dental arch characteristics, and ethnicity [4].

Interdental papilla morphology represents another important component of anterior periodontal esthetics, as incomplete papilla fill may result in visible open gingival embrasures and compromise the appearance of the maxillary anterior region [14]. Papillary characteristics can be evaluated through both their vertical dimension, represented by papilla height (PH), and their clinical fill status within the interdental space. Papilla presence and dimensions have been associated with several local anatomical factors, including the distance between the contact point and alveolar bone crest, as classically described [15], as well as tooth form and the characteristics of the interdental space [8,14]. However, whether papilla height and fill status are related to the broader soft- and hard-tissue characteristics of the periodontal phenotype remains less well understood.

Therefore, this study aimed to investigate the relationships of interdental papilla characteristics, including papilla status and PH, with GT, KTW, and buccal BT in the maxillary central incisor region. In addition, periodontal phenotype distribution and the relationships among its soft- and hard-tissue components were evaluated in young adults.

## 2. Materials and Methods

### 2.1. Study Design

This observational cross-sectional study was conducted in accordance with the principles of the Declaration of Helsinki and approved by the Ethics Committee of Cankiri Karatekin University, Health Sciences, Turkey (Approval No: 2024/13, Date: 30 April 2024). All participants provided written informed consent prior to enrollment.

### 2.2. Study Population

A priori power analysis was performed using G*Power 3.1 (Heinrich-Heine-Universität, Düsseldorf, Germany) software for a one-way ANOVA comparing morphometric parameters. A medium effect size (Cohen’s f = 0.29) was assumed based on the between-group variability in gingival width reported in a similar clinical population [16]. With an alpha level of 0.05 and a desired power of 80%, the minimum required sample size was calculated to be 120 participants, yielding an actual power of 80.86%. A total of 119 systemically and periodontally healthy individuals (84 females, 35 males; aged 18–30 years) were ultimately included.

The inclusion criteria were as follows: (1) systemically healthy individuals presenting with an intact periodontium, operationally defined according to the 2017 World Workshop consensus criteria [17] (probing depth ≤ 3 mm, no clinical attachment loss or gingival recession with the cementoenamel junction (CEJ) entirely covered), and optimal oral hygiene defined as full-mouth bleeding and plaque scores < 20% [18], with complete absence of localized bleeding on probing or visual inflammatory edema at the evaluated maxillary anterior sites; (2) radiographic confirmation of an intact alveolar bone crest and absence of apical or marginal radiolucencies on cone-beam computed tomography (CBCT) scans; (3) indication for CBCT imaging for diagnostic purposes (e.g., implant planning, impacted third molars); (4) age 18–30 years with an intact maxillary anterior dentition (all central incisors, lateral incisors, and canines present), ensuring standardized occlusal and interdental papilla assessment; (5) absence of restorations, prosthetic crowns, or orthodontic appliances in the maxillary anterior region; (6) absence of systemic diseases or medication use known to affect gingival metabolism or dimensions (e.g., calcium channel blockers, immunosuppressants, anticonvulsants); (7) absence of melanin hyperpigmentation that could impair visual optical assessment; and (8) participants of a homogeneous ethnic background to minimize anatomical variability. The exclusion criteria were: (1) pregnancy or lactation; (2) missing, fractured, or restored maxillary anterior teeth; (3) presence of gingival recession (intraoral exposure of the CEJ); (4) periodontal probing depth > 3 mm at any anterior site; (5) presence of dental implants in the anterior maxilla; and (6) current or past smoking history.

### 2.3. Clinical Evaluation

The maxillary CIs were selected as the reference teeth because differences between gingival phenotypes are most clearly expressed at this site, and because the CI region represents the most esthetically exposed and clinically challenging area of the maxillary anterior dentition [8]. Although the presence of all maxillary anterior teeth (central incisors, lateral incisors, and canines) was required for inclusion, the morphometric and gingival phenotype analyses were restricted to the maxillary CIs. Owing to technical issues, the data of one participant could not be obtained, and repeated data collection was not feasible. As the study relied on preexisting CBCT scans taken for clinical indications, recruiting an additional participant was not possible. Therefore, the study was completed with 119 participants. The unit of analysis was the individual tooth (central incisor); as both maxillary central incisors were assessed in each participant, a total of 238 central incisors were analyzed.

Two examiners were involved in this study: B.K. (periodontist), who performed all clinical measurements and participated in CBCT-based GT assessment, and Z.O. (radiologist), who participated in CBCT-based GT assessment and performed all BT measurements.

Clinical measurements were performed by a single experienced periodontist (B.K.) via a UNC-15 periodontal probe (Hu-Friedy^®^, Chicago, IL, USA). Each measurement was repeated twice to ensure consistency. The parameters recorded included the probing depth, FMPI, FMBI, KTW, PT, crown width/crown length ratio (CW/CL), and PH.

Keratinized tissue width (KTW) was measured from the midfacial margin to the mucogingival junction (nearest millimeter). The mucogingival junction was identified using the visual method, based on the color transition between the attached gingiva and the alveolar mucosa. In cases where the mucogingival junction was not clearly detectable, the “wrinkle test” was utilized [6,19].

Probe visibility was selected as a simple, non-invasive clinical approach for initial gingival phenotype categorization, consistent with the 2017 World Workshop framework [1,20].

Gingival thickness (GT) was determined by probe transparency (PT) at the midfacial sulcus:

Score 0 = probe visible (thin phenotype);

Score 1 = probe not visible (thick phenotype) [21].

CW/CL ratio represented the crown length measured from the incisal edge to the most coronal gingival margin; for crown width, the clinical crown was divided vertically into cervical, middle, and incisal thirds, and the mesiodistal distance between the proximal surfaces was recorded at the junction of the cervical and middle thirds.

Papilla height (PH) was measured to the nearest millimeter as the perpendicular distance from the papilla tip to an imaginary line connecting the gingival zeniths of the adjacent central incisors. Papilla fill status between the maxillary CIs was evaluated according to the Nordland and Tarnow classification [22]. Papillae were categorized as Normal when the interdental space was completely filled up to the contact area; Class I when the papilla tip was located apical to the contact area but coronal to the proximal CEJ; Class II when the tip was at or apical to the proximal CEJ but remained coronal to the facial CEJ; and Class III when the papilla tip was at or apical to the facial CEJ.

### 2.4. Cone-Beam Computed Tomography Evaluation

CBCT scans were acquired via a Castellini X-Rad1 US TR10 Plus device (90 kVp, 4 mA, 8 pulses/s, voxel size 0.3 mm, FOV 10 × 8 cm). During image acquisition, participants were instructed to keep their mouths full of air to displace the lips and cheeks away from the gingival tissue, creating a visible air space that facilitated soft-tissue delineation, as previously described [23]. This non-invasive technique allowed clear visualization of the gingival contour without the use of a mechanical retractor. Images were analyzed with a DICOM viewer (3D Slicer software, Slicer 5.8.1, GitHub, San Francisco, CA, USA) under standardized lighting conditions on a Lenovo 15.0 510 80SR monitor, which was used to generate three-dimensional reconstructions to establish the correct anatomical orientation and identify the standardized cross-sectional plane for each tooth. Measurements of GT and BT were then performed on two-dimensional cross-sectional slices. These cross-sectional slices were aligned parallel to the long axis of each central incisor, passing through the mid-sagittal plane of the tooth. Soft-tissue boundaries, including the gingival margin and the interface between soft tissue and the adjacent air space, were manually delineated independently by the two calibrated examiners (B.K., Z.O.) based on differences in radiographic density.

**Figure 1:**
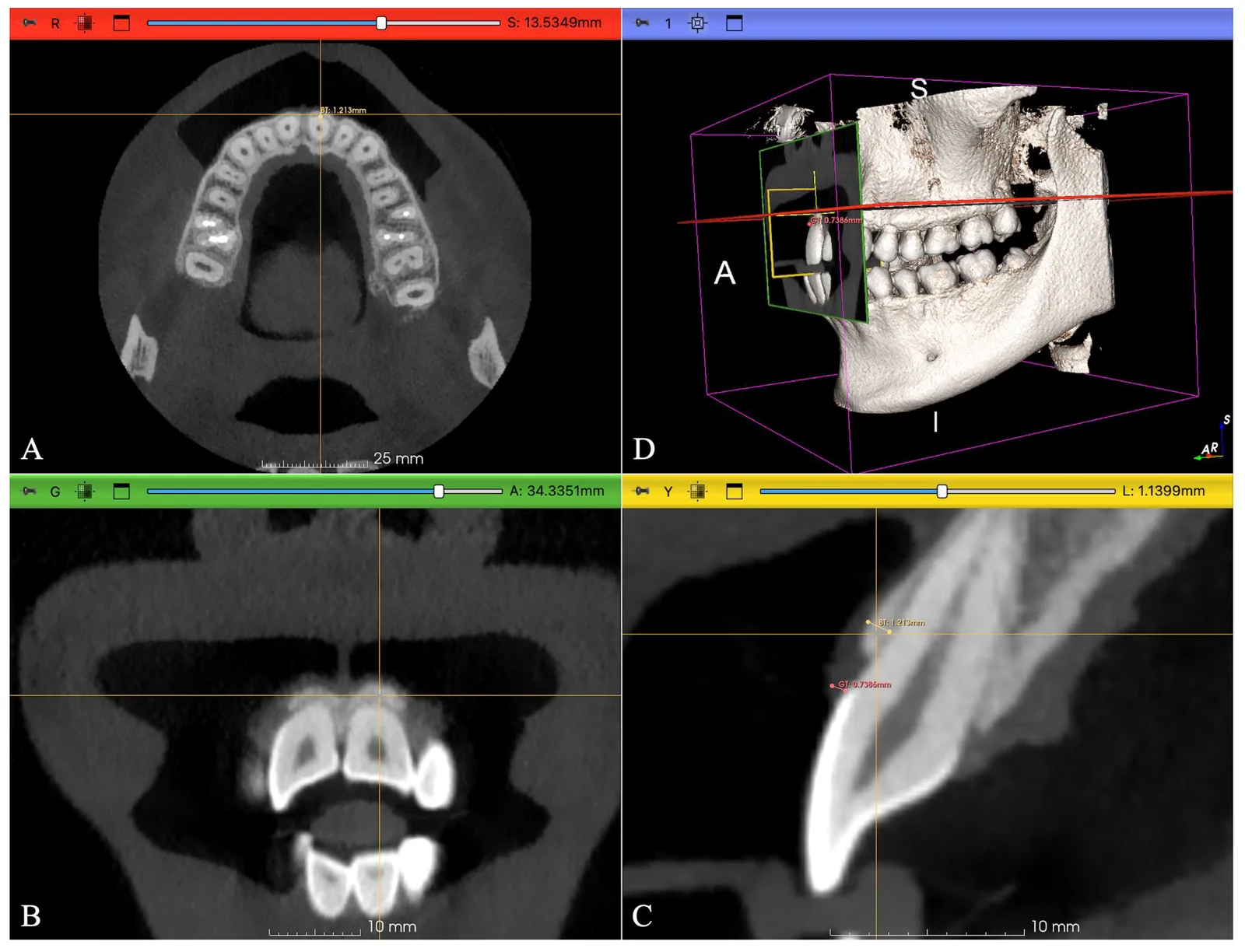
Measurement of gingival thickness and bone thickness on cross-sectional CBCT images. (**A**) Axial, (**B**) coronal, and (**C**) sagittal planes, and (**D**) three-dimensional (3D) volume-rendered reconstruction (A: anterior, S: superior, I: inferior).

GT at the CEJ was measured on cross-sectional slices (Figure 1). At this level, a GT ≤ 1.0 mm was classified as thin, and a GT > 1.0 mm was classified as thick, following Kan et al. [21]. Two independent examiners (B.K., Z.O.) performed the measurements; interexaminer agreement for phenotypic categorization was almost perfect for both teeth #11 (κ = 0.879) and #21 (κ = 0.877). Discrepancies were resolved by consensus. BT was measured at the alveolar crest (Figure 1) by a single calibrated radiologist (Z.O.). Measurements were conducted under standardized, dim ambient lighting conditions with appropriate breaks incorporated to minimize examiner fatigue. Each measurement was repeated twice, in two separate sessions 10 days apart, without reference to the first-session results. Intra-examiner reliability was assessed using the intraclass correlation coefficient (ICC), which demonstrated excellent reproducibility for both tooth #11 (ICC = 0.998, 95% CI: 0.997–0.999, *p* < 0.001) and tooth #21 (ICC = 0.997, 95% CI: 0.996–0.998, *p* < 0.001). The mean of the two measurements was used in the analysis.

### 2.5. Statistical Analysis

All the data were analyzed via the IBM SPSS Statistics Standard Concurrent User Version 27 software package (IBM Corp., Armonk, NY, USA). Descriptive statistics are presented as the frequency (n), percentage (%), mean ± standard deviation ($\bar{x}$ ± SD), median (M), minimum (min), and maximum (max) values. Cohen’s weighted kappa coefficient was used to assess the agreement among examiners. The strength of agreement was interpreted on the basis of the classification proposed by Landis and Koch: values below 0 were considered “poor agreement,” 0.00–0.20 as “slight agreement,” 0.21–0.40 as “fair agreement,” 0.41–0.60 as “moderate agreement,” 0.61–0.80 as “substantial agreement,” and 0.81–1.00 as “almost perfect agreement.” Intra-examiner reliability for continuous radiographic measurements was evaluated using the ICC with a 95% confidence interval (two-way mixed-effects model, absolute agreement). ICC values were interpreted as poor (<0.50), moderate (0.50–0.75), good (0.75–0.90), and excellent (>0.90).

For comparisons between two groups, the independent samples t test was used when the data were normally distributed, whereas the Mann–Whitney U test was applied for nonnormally distributed data. In cases involving variables with more than two categories, one-way analysis of variance (ANOVA) was conducted if normality assumptions were met; otherwise, the Kruskal–Wallis H test was employed. If a significant difference was detected via ANOVA or Kruskal–Wallis analysis, the Dunn–Bonferroni post hoc correction was used for multiple comparisons. Relationships between variables were assessed via the Spearman correlation coefficient. A *p* value of less than 0.05 was considered statistically significant.

## 3. Results

The participants had a mean age of 21.55 ± 2.15 years. Among the 119 individuals included, females represented the majority (70.6%), whereas males accounted for 29.4%. Based on CBCT-measured GT, the thin phenotype was identified in 54.6% of the evaluated sites, whereas 45.4% were classified as having a thick phenotype. Clinical assessment using PT identified a thin phenotype in 43.6% of the sites. The two approaches showed moderate agreement in phenotype classification (κ = 0.583). Interexaminer agreement for CBCT-based GT assessment was almost perfect, with κ values of 0.879 for tooth #11 and 0.877 for tooth #21. Detailed agreement statistics are presented in Table 1.

**Figure 2:**
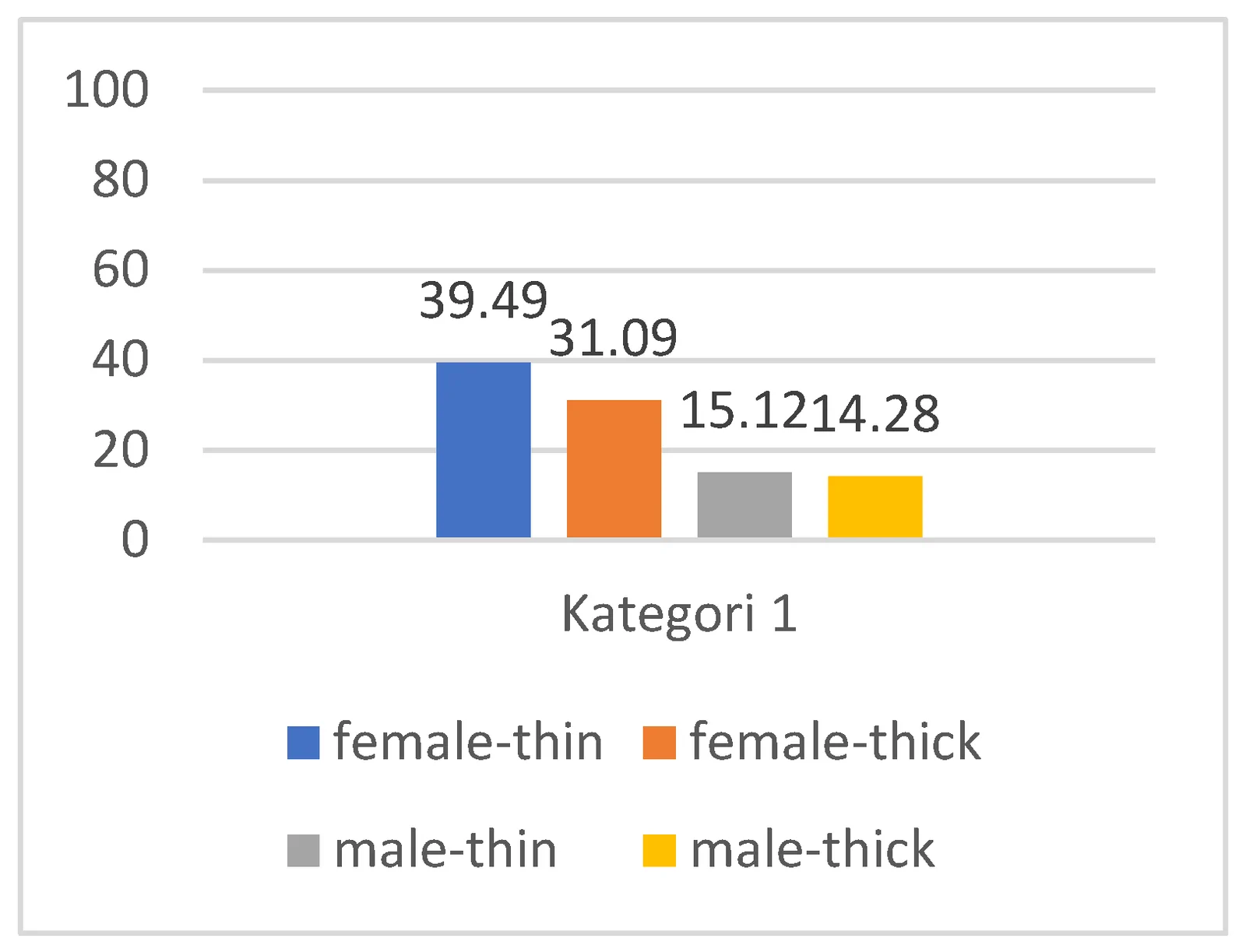
Distribution of thin and thick gingival phenotypes in male and female participants with maxillary central incisors.

The distribution of gingival phenotypes for maxillary CIs according to sex is presented in Figure 2.

The mean KTW for teeth #11 and #21 was approximately 4.5 mm. The average CL of these teeth was 9.73 mm, and the mean CW was 7.3 mm, yielding a CW/CL ratio of 0.75. Most participants (79%) presented with normal interdental papilla fill. Class I papilla loss was observed in the remaining cases, some of which were accompanied by a diastema; no Class II or Class III papilla loss was observed. Mean PH ranged from 3.7 to 4.0 mm. The mean GT and BT values obtained from CBCT measurements for teeth #11 and #21 are summarized in Table 2.

There was no statistically significant association between papilla status and the variables GT, BT, KTW, and PH (Table 3).

As shown in Table 4, a strong positive correlation was identified between GT11 and BT11 (ρ = 0.410, *p* < 0.001) and between GT21 and BT21 (ρ = 0.521, *p* < 0.001), indicating that greater GT was associated with increased buccal BT in the corresponding region. Weak but statistically significant correlations were also found between GT11 and KTW11 (ρ = 0.204, *p* < 0.05) and between GT21 and KTW21 (ρ = 0.216, *p* < 0.05), suggesting that thicker gingiva tended to accompany a slightly wider keratinized tissue zone. In addition, a strong correlation was noted between GT11 and GT21 (ρ = 0.809, *p* < 0.001), demonstrating bilateral symmetry in the GT between the right and left maxillary CIs. This symmetry implies a relatively stable and consistent gingival phenotype across the anterior maxilla, which may be clinically relevant for diagnostic and treatment planning purposes.

As presented in Table 5, no statistically significant differences were observed between the thin and thick gingival phenotype groups for KTW11, CL11, CL21, CW11, and CW21; the CW/CL ratio; age; or papilla height values between teeth 12–11, 11–21, and 21–22. In contrast, significant differences were identified for GT11, GT21, BT11, BT21, and KTW21 (*p* < 0.05).

The mean GT values for teeth #11 and #21 were greater in the thick-phenotype group (1.01–1.02 mm) than in the thin-phenotype group (0.69–0.71 mm). Similarly, BT was greater in the thick-phenotype group (BT11: 0.79 mm; BT21: 0.74 mm) than in the thin-phenotype group (BT11: 0.61 mm; BT21: 0.59 mm). The mean KTW21 was also slightly greater in the thick phenotype (4.72 mm) than in the thin phenotype (4.17 mm).

As presented in Table 6, no statistically significant differences were found between male and female participants in GT11, GT21, BT11, BT21, KTW21, the CW/CL ratio, or the PH (*p* > 0.05). In contrast, significant differences were detected for KTW11 and age (*p* < 0.05). The mean KTW at site #11 was greater in males (4.91 mm) than in females (4.46 mm), indicating a slightly wider zone of keratinized gingiva in male participants. It should be acknowledged, however, that the unequal sex distribution in the study sample may have limited the ability to identify subtle sex-related variations.

## 4. Discussion

Accurate evaluation of the periodontal phenotype is important for esthetic treatment planning, as variations in gingival and alveolar tissue dimensions may influence the outcomes of periodontal, restorative, orthodontic, and implant procedures. Beyond these phenotype-related characteristics, the interdental papilla, particularly the central papilla between the maxillary central incisors, represents another key component of anterior esthetics because of its prominent position and contribution to gingival harmony [8,10]. The principal finding of the present study was that papilla status was not significantly associated with GT, BT, KTW, or PH. In contrast, GT showed a significant positive relationship with the corresponding buccal BT, while a thin periodontal phenotype predominated in this young adult population. Together, these findings suggest that although gingival and alveolar dimensions are structurally related, papilla fill may not simply reflect the conventional soft- and hard-tissue dimensions used to characterize periodontal phenotype.

The overall number of studies exploring the correlation between periodontal phenotype and papillary morphology remains limited. Babayiğit et al. [24] reported greater PH in individuals with a thick phenotype, whereas Yin et al. [25] observed narrower papillae in participants with thin gingiva. In contrast, Olssoin et al. [19], Stein [26], and Kloukos et al. [20] reported no clear correlation between GT and papilla dimensions, while Lee [27] similarly found no association between gingival phenotype and PH between the maxillary CIs. These inconsistencies may largely reflect methodological differences, varying sample characteristics, and population-specific morphological features. More importantly, previous studies have generally focused on selected phenotype-related characteristics, while the relationship between papilla height and fill status and the combined soft- and hard-tissue dimensions of the periodontal phenotype remains less well characterized.

In the present study, papilla fill status was not significantly associated with GT, BT, KTW, or PH, and mean PH was comparable between the thin (3.85 mm) and thick (3.79 mm) phenotype groups. These findings are consistent with previous reports showing no clear relationship between gingival phenotype and papillary dimensions [19,20,26] and extend these observations by considering papilla status in relation to both soft- and hard-tissue parameters. The absence of significant associations suggests that papilla fill may not be directly explained by periodontal phenotype-related dimensions alone. Rather, papillary morphology is likely influenced by a broader combination of anatomical factors. Thus, the present findings add to the limited evidence suggesting that evaluation of papilla morphology may provide information complementary to conventional periodontal phenotype assessment in the maxillary esthetic region.

In contrast to the lack of association observed for papilla status, a significant relationship between gingival and underlying alveolar bone dimensions was evident. Previous studies have also demonstrated positive relationships between GT and buccal BT. Stein et al. [26] reported such a correlation, although their GT and BT measurements were obtained at different anatomical levels (supracrestal and subcrestal), potentially limiting comparability. Subsequent investigations supported this association, with both Cook et al. [28] and Amid et al. [9] reporting significantly greater BT in individuals with a thick phenotype, regardless of the measurement depth. However, La Rocca et al. [29], in an in vivo study of maxillary teeth, failed to confirm this relationship, likely because of methodological differences in the reference levels used for GT and BT assessment.

In the present study, significant positive correlations were observed between GT and BT at both maxillary CIs (ρ = 0.410 for tooth #11 and ρ = 0.521 for tooth #21; *p* < 0.001). The mean BT values were 0.69 ± 0.20 mm and 0.66 ± 0.17 mm for teeth #11 and #21, respectively. These values are slightly lower than the approximately 0.8–1.1 mm reported in previous CBCT-based studies [30,31,32] but support the general observation that the buccal plate in the maxillary anterior region is frequently thin [5,33]. Overall, these findings support an anatomical relationship between the soft- and hard-tissue components of the periodontal phenotype.

KTW represents another component of gingival phenotype that has been associated with GT in previous investigations. A systematic review reported mean keratinized gingival widths ranging from 2.75 to 5.44 mm in individuals with a thin phenotype and from 5.09 to 6.65 mm in those with a thick phenotype [34]. Positive relationships between GT and KTW in the maxillary anterior region have also been reported [4,6,35], with several studies demonstrating greater KTW in individuals with thicker gingiva [3,10,36]. In the present study, the mean KTW around teeth #11 and #21 was approximately 4.5 mm, and GT showed weak but significant positive correlations with KTW (ρ = 0.204 and ρ = 0.216; *p* < 0.05). Thus, although thicker gingiva tended to accompany a wider keratinized tissue zone, the weak magnitude of these correlations suggests considerable individual variability in the relationship between these soft-tissue dimensions.

Previous studies have reported considerable variation in the distribution of gingival phenotypes among different populations. While some studies have shown a predominance of the thick phenotype [25,27,37], others, especially in Asian cohorts, reported thinner phenotypes [18]. Within the Turkish population, inconsistent findings have also been documented, likely due to differences in methodology, sample size, and regional variability [3,10,36]. In the present study, the thin phenotype predominated, accounting for 54.6% of sites according to CBCT-based assessment. The mean GT values recorded in this study (0.83–0.85 mm) were slightly lower than the 0.92 mm reported by Kim et al. [38], and the approximately 1.0 ± 0.3 mm reported in another CBCT-based investigation [39]. These findings indicate relatively thin gingival tissues in the anterior maxilla of the present young adult population.

PT was used as a simple clinical approach for phenotype categorization, whereas CBCT provided quantitative assessment of GT and BT. The moderate agreement between PT- and CBCT-based classifications suggests that these approaches provide related, although not interchangeable, information on gingival phenotype. Furthermore, the inclusion of a relatively homogeneous young adult cohort (18–30 years) may have reduced potential age-related variability associated with factors such as physiological gingival recession and alveolar bone remodeling.

Sex-related differences in periodontal phenotype have also been inconsistently reported. Some studies have demonstrated greater GT in males or thinner gingiva in females [3,25], whereas others found no substantial sex-related differences in GT or phenotype distribution [10,27,32]. In the present study, GT, BT, CW/CL ratio, and PH did not differ significantly between males and females. KTW was also generally comparable, except at tooth #11, where males presented a slightly greater KTW than females. Given the unequal sex distribution in the study sample, this isolated difference should be interpreted cautiously. Crown morphology has also been proposed as a potential indicator of periodontal phenotype [4]. However, previous findings have been inconsistent, and the CW/CL ratio alone may not reliably distinguish between thin and thick phenotypes [19,24,40]. Similarly, no significant differences in CL, CW, or CW/CL ratio were observed between phenotype groups in the present study. These findings further support the concept that periodontal phenotype reflects the interaction of multiple anatomical characteristics and may not be adequately represented by individual dental dimensions alone.

From a clinical perspective, the present findings suggest that periodontal phenotype assessment and evaluation of interdental papilla morphology may provide complementary information when examining the maxillary esthetic region. The lack of association between papilla fill and gingival or buccal bone thickness suggests that papillary appearance alone may not reflect the surrounding periodontal tissue dimensions. Therefore, considering papillary morphology alongside phenotype-related tissue characteristics may provide a more comprehensive basis for esthetically demanding periodontal, restorative, orthodontic, or implant treatment planning. These findings should, however, be interpreted primarily within the young, periodontally healthy population investigated.

Several limitations should be acknowledged. The use of a convenience sample of individuals referred for CBCT imaging may have introduced selection bias. Although intraoral measurements were performed twice by a single calibrated examiner, formal reliability statistics were not calculated for clinical parameters, and the 1-mm resolution of the UNC-15 probe may have limited the detection of submillimeter variations in PH and KTW. Finally, generalizability is limited by the uneven sex distribution, the restriction of analyses to maxillary CIs, the low prevalence of advanced papilla defects, and the lack of radiographic assessment of the interdental contact-point-to-bone-crest distance.

## 5. Conclusions

Within the limitations of this study, in periodontally healthy young adults, interdental papilla status was not associated with GT, BT, KTW, or PH, despite a significant relationship between gingival and underlying buccal bone thickness. These findings suggest that papilla fill may not directly reflect conventional periodontal phenotype dimensions and support considering papillary morphology alongside soft- and hard-tissue characteristics when evaluating the maxillary esthetic region.

## Figures and Tables

**Table 1:** Interexaminer and intermethod agreement for gingival thickness measurements.

	Weighted Kappa	Asymptotic			95% Asymptotic Confidence Interval	
		*se*	*z*	*p*	Lower Bound	Upper Bound
E1.11–E2.11	0.879	0.021	14.938	**0.001**	0.838	0.920
E1.21–E2.21	0.877	0.023	15.066	**0.001**	0.832	0.922
CBCT × Probe Transparency	0.583	0.073	6.469	**0.001**	0.44	0.726

E1.11 = Gingival thickness measurement of tooth #11 by Examiner 1 (B.K.); E2.11 = Examiner 2 (Z.O.). E1.21 = Measurement of tooth #21 by Examiner 1 (B.K.); E2.21 = Examiner 2 (Z.O.). Bold values indicate statistical significance (*p* < 0.001).

**Table 2:** Descriptive statistics for GT, BT, KTW, the CW/CL ratio, and PH in maxillary central incisors.

		Mean ± SD	Median (Min.–Max.)
GT	#11	0.83 ± 0.21	0.87 (0.32–1.22)
	#21	0.85 ± 0.2	0.89 (0.32–1.24)
BT	#11	0.69 ± 0.2	0.74 (0.3–1.46)
	#21	0.66 ± 0.17	0.7 (0.3–1.15)
KTW	#11	4.6 ± 1.18	5 (2–7)
	#21	4.42 ± 1.17	4 (2–7)
CW/CL	#11–#21	0.75 ± 0.1	0.74 (0.55–1.1)
PH	#12–#11	3.68 ± 0.83	4 (2–5)
	#11–#21	3.96 ± 0.82	4 (2–6)
	#21–#22	3.84 ± 0.8	4 (2–5)

#11: right maxillary central incisor, #21: left maxillary central incisor, #12: right maxillary lateral incisor, #22: left maxillary lateral incisor.

**Table 3:** Comparison of GT, BT, KTW, and PH values according to papilla status.

	Papilla Status			t	*p*
	**Normal**	Class I	Class I (Diastema)		
GT11	0.82 ± 0.21	0.83 ± 0.22	0.9 ± 0.18	3.304	0.347 ^€^
	0.87 (0.37–1.22)	0.85 (0.32–1.1)	0.92 (0.62–1.13)		
GT21	0.85 ± 0.2	0.85 ± 0.22	0.84 ± 0.16	2.043	0.564 ^€^
	0.9 (0.42–1.24)	0.87 (0.32–1.12)	0.82 (0.61–1.08)		
BT11	0.68 ± 0.2	0.7 ± 0.19	0.75 ± 0.23	2.112	0.550 ^€^
	0.72 (0.3–1.46)	0.77 (0.32–0.91)	0.74 (0.36–1.22)		
BT21	0.65 ± 0.17	0.65 ± 0.16	0.72 ± 0.22	2.259	0.520 ^€^
	0.69 (0.3–1.02)	0.7 (0.32–0.91)	0.76 (0.38–1.15)		
KTW11	4.55 ± 1.22	4.85 ± 0.99	4.83 ± 1.03	3.410	0.333 ^€^
	5 (2–7)	5 (3–7)	5 (3–7)		
KTW21	4.39 ± 1.18	4.46 ± 1.2	4.75 ± 1.06	2.777	0.427 ^€^
	4 (2–7)	4 (3–7)	5 (3–7)		
PH 12–11	3.72 ± 0.86	3.54 ± 0.66	3.42 ± 0.67	4.305	0.230 ^€^
	4 (2–5)	3 (3–5)	3 (3–5)		
PH 11–21	3.96 ± 0.85	3.77 ± 0.6	4.08 ± 0.79	3.295	0.348 ^€^
	4 (2–6)	4 (3–5)	4 (2–5)		
PH 21–22	3.86 ± 0.83	3.77 ± 0.6	3.67 ± 0.78	3.267	0.352 ^€^
	4 (2–5)				

#11 right maxillary central incisor, #21: left maxillary central incisor, #12: right maxillary lateral incisor, #22: left maxillary lateral incisor. €: Kruskal–Wallis test.

**Table 4:** Correlations among morphometric parameters.

		GT11	GT21	BT11	BT21	KTW11	KTW21	CW/CL	PH-12–11	PH11–21
**GT21**	*rho*	0.809 *								
	*p*	<0.001								
**BT11**	*rho*	0.410 *	0.499							
	*p*	<0.001	<0.001							
**BT21**	*rho*	0.549	0.521 *	0.749						
	*p*	<0.001	<0.001	<0.001						
**KTW11**	*rho*	0.204 *	0.070	0.010	−0.087					
	*p*	0.016	0.450	0.911	0.346					
**KTW21**	*rho*	0.136	0.216 *	0.033	−0.055	0.780				
	*p*	0.139	0.018	0.721	0.554	<0.001				
**CW/CL**	*rho*	−0.115	−0.081	0.200	0.238	−0.120	−0.171			
	*p*	0.214	0.381	0.029	0.009	0.192	0.063			
**PH 12** **–** **11**	*rho*	−0.124	−0.029	0.087	0.009	0.009	0.014	0.124		
	*p*	0.179	0.754	0.349	0.923	0.920	0.883	0.181		
**PH 11–21**	*rho*	0.011	0.082	0.037	0.012	0.025	0.043	0.083	0.424	
	*p*	0.909	0.378	0.692	0.898	0.789	0.641	0.371	<0.001	
**PH 21–22**	*rho*	−0.261	−0.155	−0.059	−0.208	0.005	0.023	0.001	0.623	0.368
	*p*	0.004	0.091	0.524	0.023	0.960	0.803	0.988	<0.001	<0.001

ρ: Spearman’s correlation coefficient; * *p* < 0.05.

**Table 5:** Comparison of clinical and radiographic parameters between the thin and thick gingival phenotype groups.

	Periodontal Phenotype		t	*p*
	Thin	Thick		
GT11 Median (Min. –Max.)	0.69 ± 0.16	1.01 ± 0.1	**8** **.** **576**	**0.001** ^†^
	0.68 (0.32–1)	1.02 (0.68–1.22)		
GT21 Median (Min.–Max.)	0.71 ± 0.15	1.02 ± 0.09	**12** **.** **865**	**0.001** ^‡^
	0.71 (0.32–1.01)	1.01 (0.74–1.24)		
BT11 Median (Min.–Max.)	0.61 ± 0.21	0.79 ± 0.13	**4.932**	**0.001** ^†^
	0.63 (0.3–1.22)	0.78 (0.56–1.46)		
BT21 Median (Min.–Max.)	0.59 ± 0.18	0.74 ± 0.11	**5.400**	**0.001** ^†^
	0.6 (0.3–1.15)	0.77 (0.45–1.08)		
KTW11 Median (Min.–Max.)	4.51 ± 1.08	4.7 ± 1.3	0.705	0.481 ^†^
	5 (2–7)	5 (2–7)		
KTW21 Median (Min.–Max.)	4.17 ± 1.01	4.72 ± 1.28	**2.438**	**0.015** ^†^
	4 (3–7)	5 (2–7)		
CL11 Median (Min.–Max.)	9.62 ± 1.06	9.87 ± 1.01	1.140	0.254 ^†^
	10 (7–11)	10 (7–12)		
CL21 Median (Min.–Max.)	9.58 ± 1.07	9.91 ± 1.09	1.611	0.107 ^†^
	10 (7–11)	10 (6–12)		
CW11 Median (Min.–Max.)	7.26 ± 0.71	7.37 ± 0.9	0.529	0.597 ^†^
	7 (5–9)	7(5–9)		
CW21 Median (Min.–Max.)	7.23 ± 0.72	7.35 ± 0.87	0.700	0.484 ^†^
	7 (5–9)	7 (5–9)		
CW/CL Median (Min.–Max.)	0.76 ± 0.1	0.75 ± 0.1	0.404	0.686 ^†^
	0.74 (0.55–1)	0.75 (0.56–1.1)		
Age Median (Min.–Max.)	21.45 ± 2.27	21.67 ± 2.01	0.761	0.447 ^†^
	21 (18–30)	22 (18–26)		
PH 12–11 Median (Min.–Max.)	3.69 ± 0.86	3.67 ± 0.8	0.108	0.914 ^†^
	4 (2–5)	4 (2–5)		
PH 11–21 Median (Min.–Max.)	3.89 ± 0.75	4.04 ± 0.89	0.838	0.402 ^†^
	4 (2–5)	4 (2–6)		
PH 21–22 Median (Min.–Max.)	3.98 ± 0.76	3.67 ± 0.82	2.056	0.400 ^†^
	4 (2–5)	4 (2–5)		

#11 right maxillary central incisor, #21: left maxillary central incisor, #12: right maxillary lateral incisor, #22: left maxillary lateral incisor, †: Mann–Whitney U test; ‡: Independent samples t-test. Bold values indicate statistical significance (*p* < 0.05)

**Table 6:** Comparison of clinical parameters between male and female participants.

	Gender		t	*p*
	Female	Male		
GT11	0.83 ± 0.22	0.85 ± 0.2	0.394	0.694 ^†^
	0.86 (0.37–1.22)	0.87 (0.32–1.14)		
GT21	0.84 ± 0.19	0.87 ± 0.22	0.767	0.443 ^†^
	0.89 (0.42–1.2)	0.9 (0.32–1.24)		
	0.88 (0.41–1.12)	0.89 (0.4–1.17)		
BT11	0.7 ± 0.19	0.66 ± 0.22	0.359	0.720 ^†^
	0.73 (0.3–1.46)	0.74 (0.32–1.08)		
BT21	0.67 ± 0.15	0.63 ± 0.21	0.972	0.331 ^†^
	0.7 (0.32–1.15)	0.65 (0.3–1.08)		
KTW11	4.46 ± 1.08	4.91 ± 1.36	**2.099**	**0.036** ^†^
	5 (2–7)	5 (2–7)		
KTW21	4.33 ± 1.12	4.63 ± 1.26	1.118	0.264 ^†^
	4 (3–7)	5 (2–7)		
CW/CL	0.75 ± 0.1	0.76 ± 0.1	0.121	0.904 ^†^
	0.78 (0.56–1)	0.73 (0.55–1.1)		
Age	21.31 ± 2.21	22.11 ± 1.92	**2.239**	**0.025** ^†^
	21 (18–30)	22 (18–26)		
PH 12–11	3.7 ± 0.89	3.63 ± 0.69	0.436	0.663 ^†^
	4 (2–5)	4 (3–5)		
PH 11–21	3.88 ± 0.81	4.14 ± 0.81	1.559	0.119 ^†^
	4 (2–5)	4 (2–6)		
PH 21–22	3.87 ± 0.83	3.77 ± 0.73	0.679	0.497 ^†^
	4 (2–5)	4 (2–5)		

#11: right maxillary central incisor, #21: left maxillary central incisor, #12: right maxillary lateral incisor, #22: left maxillary lateral incisor, †: Mann–Whitney U test. Bold values indicate statistical significance (*p* < 0.05)

## Data Availability

The data that support the findings of this study are available from the corresponding author upon reasonable request. Individual CBCT and clinical measurement datasets are not publicly available due to privacy and ethical restrictions.

## Abbreviations

The following abbreviations are used in this manuscript:

GT	Gingival thickness
KTW	Width of keratinized tissue
CIs	Central incisors
BT	Bone thickness
CBCT	Cone-beam computed tomography
PT	Probe transparency
FMPI	Full Mouth Plaque Index
FMBI	Full Mouth Bleeding Index
CEJ	Cemento-enamel junction
CW/CL	Crown width/crown length ratio
PH	Papilla height
ICC	Intraclass correlation coefficient
